# Cytoplasmic PPARγ is a marker of poor prognosis in patients with Cox-1 negative primary breast cancers

**DOI:** 10.1186/s12967-020-02271-6

**Published:** 2020-02-21

**Authors:** Wanting Shao, Christina Kuhn, Doris Mayr, Nina Ditsch, Magdalena Kailuwait, Verena Wolf, Nadia Harbeck, Sven Mahner, Udo Jeschke, Vincent Cavaillès, Sophie Sixou

**Affiliations:** 1 Breast Center, Department of Obstetrics and Gynecology, University Hospital, LMU Munich, Munich, Germany; 2 Department of Pathology, LMU Munich, Munich, Germany; 3 IRCM-Institut de Recherche en Cancérologie de Montpellier, INSERM U1194, Université Montpellier, Parc Euromédecine, 208 rue des Apothicaires, 34298 Montpellier Cedex 5, France; 4 Faculté des Sciences Pharmaceutiques, Université Paul Sabatier Toulouse III, 31062 Toulouse Cedex 09, France; 5 Cholesterol Metabolism and Therapeutic Innovations, Cancer Research Center of Toulouse (CRCT), UMR 1037, CNRS, Inserm, UPS, Université de Toulouse, 31037 Toulouse, France

**Keywords:** PPARγ, Cytoplasmic, Cox-1, Cox-2, Overall survival, Breast cancer

## Abstract

**Background:**

The aim of this study was to investigate the expression of the nuclear receptor PPARγ, together with that of the cyclooxygenases Cox-1 and Cox-2, in breast cancer (BC) tissues and to correlate the data with several clinicobiological parameters including patient survival.

**Methods:**

In a well characterized cohort of 308 primary BC, PPARγ, Cox-1 and Cox-2 cytoplasmic and nuclear expression were evaluated by immunohistochemistry. Correlations with clinicopathological and aggressiveness features were analyzed, as well as survival using Kaplan–Meier analysis.

**Results:**

PPARγ was expressed in almost 58% of the samples with a predominant cytoplasmic location. Cox-1 and Cox-2 were exclusively cytoplasmic. Cytoplasmic PPARγ was inversely correlated with nuclear PPARγ and ER expression, but positively with Cox-1, Cox-2, and other high-risk markers of BC, e.g. HER2, CD133, and N-cadherin. Overall survival analysis demonstrated that cytoplasmic PPARγ had a strong correlation with poor survival in the whole cohort, and even stronger in the subgroup of patients with no Cox-1 expression where cytoplasmic PPARγ expression appeared as an independent marker of poor prognosis. In support of this cross-talk between PPARγ and Cox-1, we found that Cox-1 became a marker of good prognosis only when cytoplasmic PPARγ was expressed at high levels.

**Conclusion:**

Altogether, these data suggest that the relative expression of cytoplasmic PPARγ and Cox-1 may play an important role in oncogenesis and could be defined as a potential prognosis marker to identify specific high risk BC subgroups.

## Background

Breast cancer (BC), the most commonly diagnosed malignant tumor in women, is also the most frequent cause of cancer death worldwide [1] and a significant global public health problem. BC is highly heterogeneous in its pathological characteristics, which raised a tremendous challenge for treatment selection [2]. So far, few biomarkers have been well recognized in invasive breast carcinomas, including estrogen receptor (ER) and progesterone receptor (PR), which are associated with a better outcome and are predictive of endocrine sensitivity. Overexpression of human epidermal growth factor receptor 2 (HER2) is related with decreased relapse-free survival (RFS) and overall survival (OS) [3, 4]. Agents targeting ER and HER2, such as tamoxifen and trastuzumab, have been very successful as BC therapeutics. However, multifaceted mechanisms emerged in tumors, causing resistance to endocrine treatment in single or combination therapies [5]. Thus, comprehensive identification of more biomarkers and molecular targets is essential for optimal and personalized clinical BC management.

Peroxisome proliferator-activated receptors (PPARs) belong to the nuclear receptor (NR) superfamily [6] and function as ligand-activated transcription factors [7]. Following activation by ligands (e.g. 15d-PGJ_2_ or the synthetic ligand thiazolidinedione), PPARs heterodimerize with retinoid X receptor (RXR) and interact with proliferator-activated receptor response elements (PPREs) present in target gene promoters [8]. Although the NR superfamily was defined due to genomic actions of the receptors which require nuclear localization, it has been suggested that PPARs localize first in the cytoplasm with specific associated functions [9].

Among the three PPAR isoforms (α, β/δ and γ), PPARγ plays a crucial role in adipogenesis and lipid metabolism [10] and is also found expressed in many human cancers, including BC [11]. PPARγ influences inflammatory processes, cell proliferation, differentiation, apoptosis and tumor angiogenesis [10, 12]. A tumor promoting effect of PPARγ has been reported in some tumors, such as liver [13], cancer [14] or colon cancer [15]. In addition, most of previous studies have revealed that PPARγ acts as a tumor suppressor in BC, inhibiting cell proliferation and inducing apoptosis in different in vivo and in vitro models [16–18]. Besides, PPARγ has been suggested as being involved in chemotherapy resistance of TNBC [19].

Interestingly, some of the PPARγ ligands, prostaglandins (PGs) are produced from the conversion of arachidonic acid by the cyclooxygenases Cox-1 and Cox-2. Cox-1 is constitutively expressed in many normal cells, whereas Cox-2 is generally considered being induced by inflammatory cytokines and growth factors, performing a significant role in carcinogenesis [20, 21]. Studies of Cox importance in tumor progression and invasion were mainly focused on the influence of Cox-2 [22]. However, it was demonstrated that Cox-1 is highly expressed and plays a pivotal role in some carcinomas, such as ovarian [23] and breast cancers [24]. More recently, Cox-1 mRNA and protein levels have been shown to be higher in malignant breast tumors than in normal tissues, whereas Cox-2 mRNA level was lower in malignant tumors. Nonetheless, stromal and glandular Cox-2 immunostaining showed higher levels in malignant breast tumors [25].

It appears therefore obvious that more attention is needed to analyze the relevance of combined expression of PPARγ and Cox (especially Cox-1) in BC. In the present study, we have analyzed expression of PPARγ and of the two Cox proteins in 308 primary BC specimens in relation to survival, to determine if either one could, independently or in relation to the others, be linked to BC progression.

## Methods

### Patient cohort

A total of 308 formalin-fixed paraffin-embedded primary BC tissues from 303 patients (5 of them are bilateral BC) who received surgeries between 2000 and 2002 at the Department of Obstetrics and Gynecology of the Ludwig-Maximilians-University Munich, Germany were collected. Local and systemic therapy treatment was given according to the guidelines at the time of diagnosis. This study was approved by the Ethical Committee of the Medical Faculty, Ludwig-Maximilian-University, Munich, Germany (approval number 048-08) and informed consent for nuclear factor analysis was obtained from all patients who were alive at the time of follow-up. Data, such as age, histological grade, metastases, local recurrence, progression, and survival were retrieved from the Munich Cancer Registry and anonymized and encoded during statistical analysis and experiments. All tumors were assessed according to UICC TNM classification, containing tumor size and extent of tumors (primary tumor size, or pT, classified as: pT1a-c, pT2, pT3, pT4a-d), lymph node status (N), and presence or absence of metastasis (M). Tumor grade was determined by an experienced pathologist (Dr. D. Mayr) of the Department of Pathology of the LMU, according to a modification of Elston and Ellis grading proposed by Bloom and Richardson [26]. Sixty (19.48%) of the 303 primary BC patients, became metastatic during the follow-up. ER, PR, HER2, Ki-67 and histological status were all determined by an experienced pathologist of the LMU Department of Pathology, as described below. HER2 2+ scores were further evaluated through fluorescence in situ hybridization (FISH) testing.

### Immunohistochemistry (IHC)

Expression of ERα, PR, and HER2 was determined at diagnosis in all BC samples of this cohort at the LMU Department of Pathology, Germany. ERα and PR expression were evaluated by immunohistochemistry, as described previously [26]. Samples showing nuclear staining in more than 10% of tumor cells were considered as hormone receptor-positive, in agreement with the guidelines at the time of the analysis (2000–2002). HER2 expression was analyzed using an automated staining system (Ventana; Roche, Mannheim, Germany), according to the manufacturer’s instructions. Ki-67 was stained using an anti-Ki67 monoclonal antibody (Dako, Hamburg, Germany) at a dilution of 1:150 on a VENTANA^®^-Benchmark Unit (Roche, Mannheim, Germany) as previously described [27]. The Ki-67 cut-off used to differentiate luminal A from luminal B tumors (all HER2 negative) was 14% as this was commonly used at the time of the analysis, although 20% is now preferred [28]. Data on N-cadherin and CD133 expression in these BC samples were extracted from a previously published study [29]. For PPARγ, Cox-1 and Cox-2 analysis by IHC, samples were processed as previously described [30, 31]. Briefly, sections were first cut and prepared from paraffin-embedded BC samples using standard protocols. Phosphate buffered saline (PBS) was used for all washes and sections were incubated in blocking solution (ZytoChem Plus HRP Polymer System Kit, ZYTOMED Systems GmbH, Berlin, Germany) before incubation with primary antibodies. All primary antibodies were rabbit IgG polyclonal used at a 1:100 dilution for 16 h at 4 °C: anti-PPARγ (ab59256, Abcam, Cambridge, UK) or anti-Cox-1 (HPA002834) and anti-Cox-2 (SAB4502491, both Sigma-Aldrich, Saint Louis, MO, USA). After incubation with a biotinylated secondary anti-rabbit IgG antibody, and with the associated avidin–biotin–peroxidase-complex (both Vectastain Elite ABC Kit; Vector Laboratories, Burlingame, CA, USA), visualization was performed with substrate and chromogen 3,3-diamino-benzidine (DAB; Dako, Glostrup, Denmark). Negative and positive controls were used to assess the specificity of the immunoreactions. Negative controls (colored in blue) were performed in BC tissue by replacement of the primary antibodies by species-specific (rabbit) isotype control antibodies (Dako, Glostrup, Denmark). Appropriate positive controls (placenta samples) were included in each experiment. Sections were counterstained with acidic hematoxylin, dehydrated and immediately mounted with Eukitt (Merck, Darmstadt, Germany) before manual analysis with a Diaplan light microscope (Leitz, Wetzlar, Germany) with 25× magnification. Pictures were obtained with a digital CCD camera system (JVC, Tokyo, Japan). All slides were analyzed by two or three independent examiners.

### Immunoreactive score (IRS)

The expression of PPARγ, Cox-1 and Cox-2 was assessed according to the immunoreactive score (IRS), determined by evaluating the proportion of positive tumor cells, scored as 0 (no staining), 1 (≤ 10% of stained cells), 2 (11–50% of stained cells), 3 (51–80% of stained cells) and 4 (≥ 80% of stained cells), and the intensity of their staining, graded as 0 (negative), 1 (weak), 2 (moderate) and 3 (strong) (IRS = percentage score × intensity score). Thus, the range of IRS value is from 0 to 12. As previously described for LCoR and RIP140 [31] and for AhR [32], PPARγ cytoplasmic and nuclear staining were evaluated in parallel, with a separate determination of cytoplasmic IRS and nuclear IRS. Total IRS was calculated by addition of cytoplasmic and nuclear IRS. For all other markers, staining and IRS were determined in the whole cells, without differentiation of nuclear and cytoplasmic staining.

### Survival and statistical analysis

Receiver operating characteristic curve (ROC) analyses were performed to calculate the optimal cut-off values between low and high PPARγ, Cox-1 and Cox-2 expressions, based upon the maximal differences of sensitivity and specificity. The threshold determined regarding OS were an IRS ≥ 3.5 for either total or cytoplasmic PPARγ, ≥ 0.5 for nuclear PPARγ and for Cox-1, and finally ≥ 1.5 for Cox-2. These thresholds were used to determine the percentages of tumors expressing low or high PPARγ, Cox-1 and Cox-2 levels described in Table 2, besides the OS analysis detailed below. To present the mean immunoreactivity levels described by the IRS in Table 2, the groups were divided into low- vs. high-expressing cases for total and cytoplasmic PPARγ, Cox-2, or into not expressing vs. expressing cases for nuclear PPARγ, Cox-1 (cut-off values of 0.5).

**Fig. 1:**
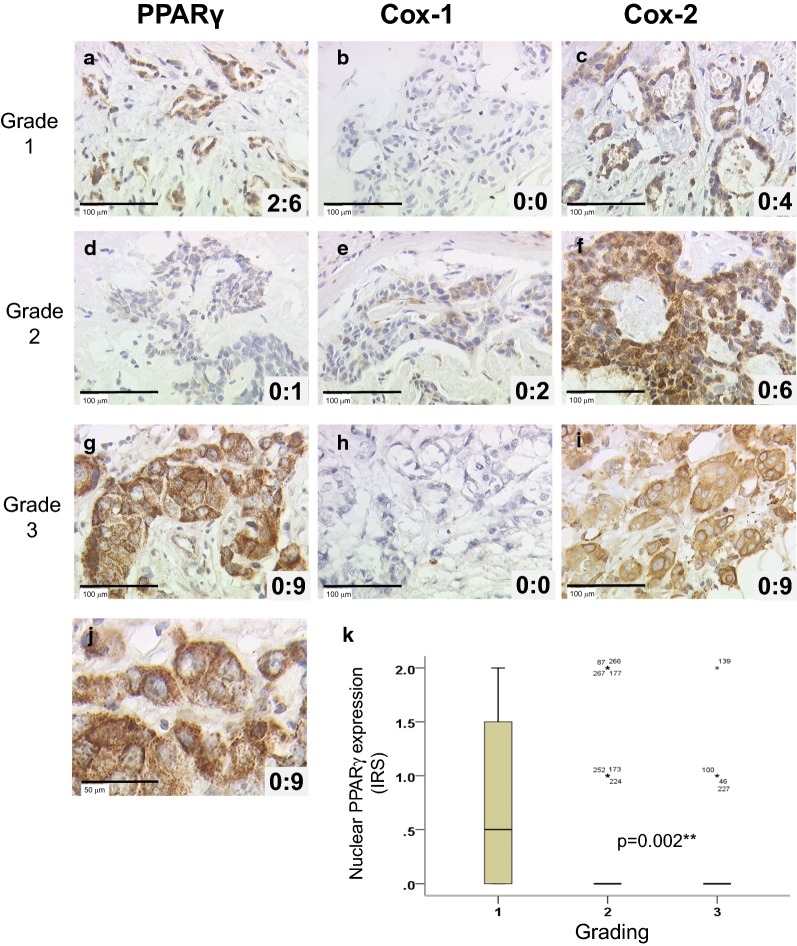
Immunohistochemical stainings of PPARγ, Cox-1 and Cox-2 expression in breast carcinoma of 3 patients and a box-plot of nuclear PPARγ and Grading. PPARγ (**a**, **d**, **g**), Cox-1 (**b**, **e**, **h**) and Cox-2 (**c**, **f**, **i**) stainings are illustrated for patients with different grading (Grade 1 in **a**–**c**, 2 in **d**–**f** and 3 in **g**–**i**), with examples of null, average or high expressions. Nucleo:cytoplasmic IRS ratios are indicated in each photomicrograph (×25 magnification) and scale bar equals 100 μm. An enlargement of **g** (high cytoplasmic and null nuclear PPARγ staining) is presented (**j**) and scale bar equals 50 μm. Correlation between nuclear PPARγ and grading was presented as box plot (**k**). The boxes represent the range between the 25th and 75th percentiles with a horizontal line at the median. The bars on top and below depict the 5th and 95th percentiles. Values more than 1.5 box lengths from the 75th percentile are indicated by circles (none) and values more than 3.0 box lengths from the 75th percentile are indicated by asterisks. The numbers on asterisks represent the case number. Statistical significance is shown as p-value from Kruskal–Wallis test (**p < 0.01)

Differences in nuclear PPARγ expression among three or more groups (Fig. 1, panel k) were tested using the non-parametric Kruskal–Wallis rank-sum test. Correlation analyses presented in Tables 3 and 4 were performed by calculating the Spearman’s-Rho correlation coefficient (p values of Spearman’s-Rho test presented). Survival times were compared by Kaplan–Meier graphics and differences in OS (or RFS) were tested for significance by using the Chi-square statistics of the log rank test. Data were assumed to be statistically significant in the case of p-value < 0.05. Kaplan–Meier curves and estimates were then provided for each subgroup and each marker. The p value and the number of patients analyzed in each subgroup are given for each chart.

Multivariable analysis for outcome (OS) presented in Table 5 was performed using the Cox regression model, and included cytoplasmic PPARγ expression and relevant clinicopathological characteristics as independent variables. Variables were selected based on theoretical considerations and forced into the model. p values and hazard ratios were indicated, knowing that the hazard ratios of covariates are interpretable as multiplicative effects on the hazard, and holding the other covariates constant.

Statistical analyses were performed using SPSS 24 (IBMSPSS Statistics, IBM Corp., Armonk, NY, USA). For all analyses, p values below 0.05 (*), 0.01 (**), or 0.001 (***) were considered statistically significant.

## Results

### PPARγ and Cox expression in breast cancers

The total cohort consisted of 308 samples from 303 primary BC (Table 1). Median age of initial diagnosis was 57.98 years (range 26.66–94.62 years) and median follow-up time was 125 months (range 0–153 months). During this period, 41 (13.3%) and 60 (19.5%) cases experienced local recurrence and distant metastasis respectively, and 90 (29.2%) women died.

**Table 1:** Clinical and pathological characteristics of all patients

Clinical and pathological characteristics^a^	N = 308^b^	%
Age, median (years)	57.98	
Follow up, average (months)	109.89	
Median	125	
Histology^c^		
Invasive lobular	41	13.31
Invasive medullar	10	3.25
Invasive mucinous	3	0.97
No special type (NST)	161	52.27
DCIS with NST	78	25.33
Unknown	15	4.87
ER status		
Positive	248	80.52
Negative	58	18.83
Unknown	2	0.65
PR status		
Positive	178	57.79
Negative	128	41.56
Unknown	2	0.65
HER2 status		
Positive	35	11.36
Negative	271	87.99
Unknown	2	0.65
Molecular subtype		
Luminal A (Ki-67 ≤ 14%)	170	55.19
Luminal B (Ki-67 > 14%)	63	20.45
HER2 positive luminal	27	8.77
HER2 positive non luminal	8	2.60
Triple negative	38	12.34
Unknown	2	0.65
Grade		
I	15	4.87
II	102	33.12
III	45	14.61
Unknown	146	47.40
Tumor size		
pT1	191	62.01
pT2	87	28.25
pT3	4	1.30
pT4	12	3.90
Unknown	14	4.55
Lymph node metastasis		
Yes	126	40.91
No	163	52.92
Unknown	19	6.17
Local recurrence^d^		
Yes	41	13.31
No	253	82.14
Unknown	14	4.55
Distant metastases^e^		
Yes	60	19.48
No	234	75.97
Unknown	14	4.55

^a^All information given refer to the primary tumor

^b^5 of 303 patients are bilateral primary BC, so we deal with the tumor as individual one (n = 308)

^c^NST include the formerly called “Invasive ductal” and “other” types

^d^Local recurrence has been detected during the follow-up of 40 patients (1 of them are bilateral BC, so n = 41)

^e^Distant metastasis has been detected during the follow-up of 58 patients (2 of them are bilateral BC, so n = 60)

The expression of PPARγ, Cox-1 and Cox-2 was analyzed by IHC staining, as illustrated in Fig. 1 for 3 patients with Grade 1 (A, B, C), 2 (D, E, F) and 3 (G, H, I) tumors. PPARγ expression (A, D, G) was present both in the nucleus and in the cytoplasm, while Cox-1 and Cox-2 (B, E, H, and C, F, I respectively) were exclusively cytoplasmic. The nucleo:cytoplasmic IRS ratios are presented in each panel (panel J shows the enlargement of PPARγ staining shown in panel G). From now, all Cox-1 and Cox-2 expression refers to their unique cytoplasmic expression, with Cox-1 staining being much fainter than Cox-2 staining, as described in Table 2. As demonstrated in the panel K, nuclear PPARγ exhibited a statistically different expression according to grading, with an inverted correlation (p = 0.002). This correlation is illustrated by focusing on the nuclear PPARγ expression observed in panels A, D and G of Fig. 1 (IRS of 2, 0 and 0 respectively) for patients with respectively grade 1, 2 and 3 tumors.

**Table 2:** Distribution of expression of PPARγ, Cox-1 and Cox-2

	PPARγ			Cox-1	Cox-2
	Total	Nuclear	Cytoplasmic		
n	262	262	262	297	285
Mean IRS ± SE	4.37 ± 0.17	0.27 ± 0.04	4.09 ± 0.17	0.34 ± 0.04	5.19 ± 0.19
IRS range	12	4	12	4	12
IRS cut-off	3.5	0.5	3.5	0.5	1.5
Number of samples with negative/low expression	111 (42.4%)	213 (81.3%)	124 (47.3%)	224 (75.4%)	36 (12.6%)
Number of samples with positive/high expression	151 (57.6%)	49 (18.7%)	138 (52.7%)	73 (24.6%)	249 (87.4%)

IRS cut-offs were defined by performing a ROC-curve analysis for DFS. The cut-off of 0.5 for nuclear PPARγ and for Cox-1 stainings, related to the low expression level of both markers in our cohort, define negative and positive expressions, instead of low and high expressions sub-groups respectively

As presented in Table 2, the mean IRS of total and cytoplasmic PPARγ expression were 4.37 and 4.09 respectively, while it was 0.27 for nuclear PPARγ. It clearly appears that, in our cohort, PPARγ expression is dramatically higher (15-fold) in the cytoplasm than in the nucleus, with maximal IRS values of 12 and 4 respectively. This is exemplified in Fig. 1 with cytoplasmic PPARγ IRS values of 1, 6 and 9, and nuclear PPARγ IRS values of 0 and 2 (panels A, D and J). IRS cut-offs were defined by performing a ROC-curve analysis for OS. Of note, the IRS cut-off of 0.5 generated for nuclear PPARγ staining is related to the low expression level of this marker in our cohort, and create sub-groups with negative vs. positive expression, instead of low vs. high expression for other cut-off values. Considering cytoplasmic or total expression of PPARγ being high for IRS value > 3.5, the high expression group is predominant in both cases (52.7 and 57.6% respectively). Only 20 patients out of 262 (7.6%) had no cytoplasmic PPARγ expression (IRS = 0), demonstrating the predominant cytoplasmic expression of PPARγ (92.4% of the tumors).

Besides, the mean IRS of cytoplasmic expression were 0.34 and 5.19 for Cox-1 and Cox-2 respectively. This is again exemplified in Fig. 1 with Cox-1 IRS values of 0 and 2 (panels B, E and H) and Cox-2 IRS values of 4, 6 and 9 (panels C, F and I), for the same 3 selected patients. Similarly to nuclear PPARγ, Cox-1 mean IRS being very low, a cut-off of 0.5 was generated, with sub-groups of negative vs. positive expression, instead of low vs. high expression for Cox-2. In our cohort, 75.4% of the samples were then Cox-1 negative, whereas the samples with a high expression of Cox-2 represented 87.37% of the cases (cut-off of 1.5). Regarding nuclear PPARγ, only 49 samples were positive (18.7%) while for Cox-1, only 73 samples (24.6%) were positive (with maximum IRS of 4 for both markers).

### Correlation between PPARγ and Cox expression

The correlations between the expression levels of PPARγ (total, nuclear and cytoplasmic), Cox-1 and Cox-2 were analyzed (Table 3). Cytoplasmic PPARγ expression exhibited a strong and significant positive correlation with total PPARγ, and a negative one with nuclear PPARγ. By contrast, nuclear and total expression of PPARγ were not correlated together. Regarding Cox expression, Cox-1 and Cox-2 levels were not correlated. Nonetheless, both Cox-1 and Cox-2 expression were significantly correlated with cytoplasmic and total PPARγ expressions. Besides, nuclear PPARγ was significantly negatively correlated with Cox-2 (and not with Cox-1).

**Table 3:** Correlation between PPARγ, Cox-1 and Cox-2 expression

n = 254 to 297	PPARγ			Cox-1	Cox-2
	Total	Nuclear	Cytoplasmic		
PPARγ					
Total	1.000				
Nuclear	0.037	1.000			
Cytoplasmic	0.959**	− 0.215**	1.000		
Cox-1	0.179**	− 0.117	0.201**	1.000	
Cox-2	0.261**	− 0.124*	0.293**	0.054	1.000

Correlations are statistically significant for p < 0.05 (*) or p < 0.01 (**), using Spearman-Rho-Test

### Correlation between PPARγ, Cox expression and clinicopathological parameters or aggressiveness markers

We then analyzed the correlations between PPARγ or Cox expression and known clinicopathological characteristics (Table 4). We also quantified the expression of two aggressiveness markers, CD133, a widely used marker for isolating cancer stem cell (CSC) [33, 34], and N-cadherin, a well-known marker for epithelial-to-mesenchymal transition (EMT) [35]. Considering first nuclear PPARγ, significant negative correlations were observed with grade (as already illustrated in Fig. 1k, and by the 3 selected patients in Fig. 1), HER2 and N-cadherin, as well as Cox-2 (as already shown in Table 3). On the contrary, total and cytoplasmic PPARγ were strongly positively correlated with HER2, CD133 and N-cadherin. Only cytoplasmic PPARγ was negatively correlated to ER. Besides, Cox-1 was positively correlated with HER2, CD133, and N-cadherin, while Cox-2 was positively correlated with Ki-67, CD133, and N-cadherin. Only Cox-1 was statistically negatively correlated with lymph node status (LNM), and only Cox-2 was positively correlated with the proliferation marker Ki-67.

**Table 4:** Correlation between PPARγ, Cox-1 and Cox-2 expression and clinicopathological or aggressiveness related parameters

	PPARγ n = 143 to 262			Cox-1	Cox-2
	Total	Nuclear	Cytoplasmic	n = 159 to 297	n = 153 to 285
Age	0.004	− 0.050	0.002	0.041	− 0.015
pT	0.118	− 0.037	0.113	− 0.049	− 0.066
pN	0.069	0.023	0.065	− 0.125*	− 0.043
Grade	0.007	− 0.205*	0.054	− 0.007	− 0.062
ER	− 0.119	0.117	− 0.142*	0.009	0.039
PR	− 0.048	0.038	− 0.049	− 0.018	0.012
HER2	0.157**	− 0.127*	0.173**	0.137*	0.090
Triple negative	0.076	− 0.062	0.085	− 0.043	− 0.052
Ki-67	0.116	− 0.039	0.119	0.084	0.155*
Focality	0.043	0.074	0.016	− 0.048	− 0.028
CD133	0.221**	− 0.007	0.230**	0.132*	0.378**
NCAD	0.412**	− 0.196**	0.447**	0.241**	0.461**

Correlations are statistically significant for p < 0.05 (*) or p < 0.01 (**), using Spearman-Rho-Test

### Correlation between PPARγ, Cox expression, and patient survival

**Fig. 2:**
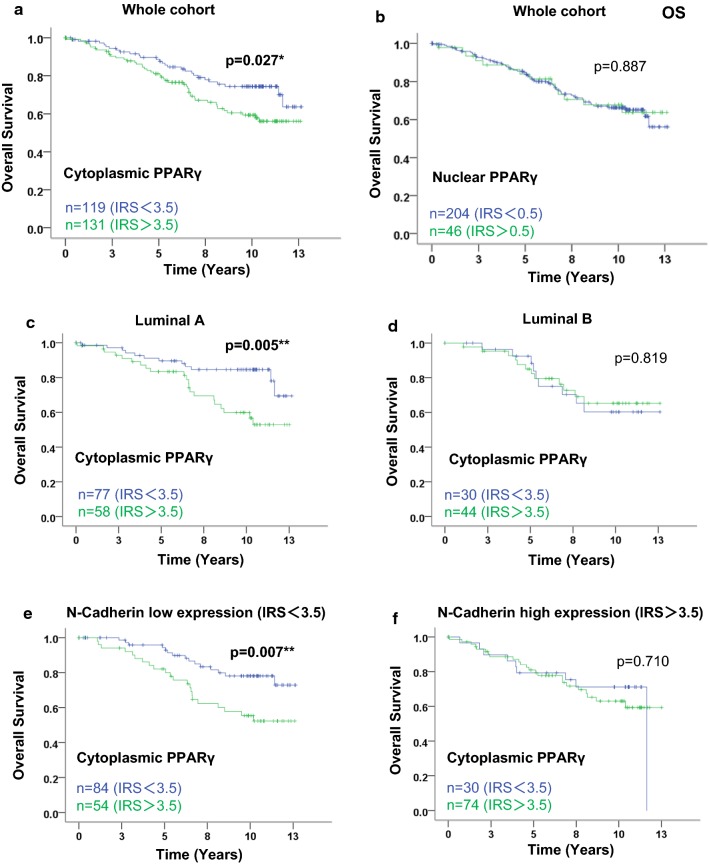
Kaplan–Meier analysis of patient overall survival according to nuclear and cytoplasmic PPARγ expression in the whole cohort, and to cytoplasmic PPARγ expression in subgroups. In the whole cohort, overall survival (OS) curves are presented according to cytoplasmic PPARγ (**a**) and nuclear PPARγ (**b**) status. In luminal (**c**, **d**) and N-Cadherin (**e**, **f**) subgroups, overall survival curves are presented according to cytoplasmic PPARγ status. The IRS cut-off values with the number of cases for each group are indicated in each graph. Statistical significance is shown as p-value from log-rank test (*p < 0.05; **p < 0.01)

In order to analyze the correlation between PPARγ and survival, we performed Kaplan–Meier analyses. We used the cut-off IRS values determined by ROC-curve analysis, allowing the maximal difference of sensitivity and specificity (as described in Table 2). In Fig. 2, considering the OS of the whole cohort, the cytoplasmic PPARγ expression was able to discriminate high expressing tumors with a significantly worse survival than patients with low expressing tumors (mean OS: 10.55 years *vs* 9.44 years, p = 0.027; Fig. 2a). On the contrary, neither nuclear PPARγ (Fig. 2b) nor total PPARγ (Additional file 1: Figure S1A) had any significant correlation with OS.

RFS analysis were performed in parallel for total, cytoplasmic and nuclear PPARγ expression (Additional file 1: Figure S1B–D respectively). Both total and cytoplasmic PPARγ significantly discriminated patients with worse RFS (when PPARγ was highly expressed) from those having better survival when PPARγ expression was low (mean RFS: 9.37 years vs 6.88 years, p = 0.001, and mean RFS: 9.30 years vs 6.70 years, p = 0.000217).

We then looked at the association between cytoplasmic PPARγ expression and OS in different subgroups by stratifying the cohort, according to parameters mentioned in Table 4. Compared to the correlation of cytoplasmic PPARγ expression with OS in the whole cohort (p = 0.027, Fig. 2a), the correlation was stronger in the subgroup of luminal A tumors (p = 0.005 Fig. 2c), and lost in the luminal B subgroup (Fig. 2d). Similarly, the correlation was very strong in the subgroup of N-Cadherin low expressing tumors (p = 0.007, Fig. 2e) and absent in the N-Cadherin high expressing tumors (Fig. 2f).

**Fig. 3:**
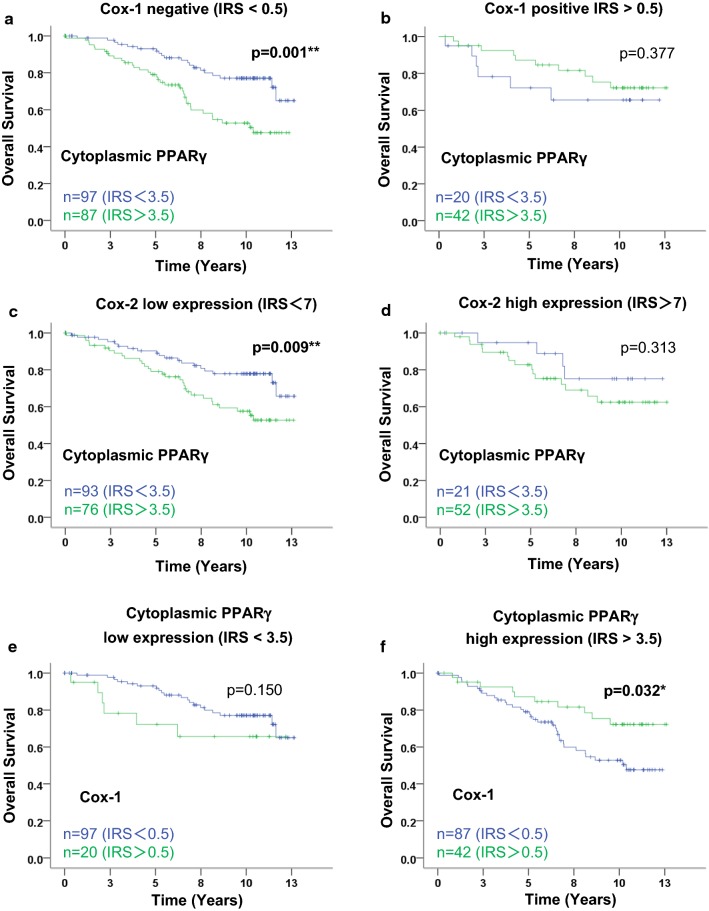
Kaplan–Meier analysis of patient overall survival according to cytoplasmic PPARγ and of Cox-1 expression in subgroups. Overall survival (OS) curves are presented according to cytoplasmic PPARγ status in Cox-1 (**a**, **b**) and Cox-2 (**c**, **d**) subgroups. OS of Cox-1 is then presented according to cytoplasmic PPARγ (**e**, **f**). The IRS cut-off values with the number of cases for each group are indicated in each graph. Statistical significance is shown as p-value from log-rank test (*p < 0.05; **p < 0.01)

We then focused on subgroups of patients according to Cox expression in their tumors. As demonstrated in Fig. 3, expression of cytoplasmic PPARγ was still clearly related to a worse prognosis in the subgroup of tumors expressing no Cox-1 (p = 0.001, Fig. 3a), as observed in the whole cohort (p = 0.027, Fig. 2a). On the contrary, no correlation of cytoplasmic PPARγ existed with the OS of patients with tumor expressing Cox-1, and the trend, although not significant, was even inverted with an apparently better prognosis for group with high cytoplasmic PPARγ expression (Fig. 3b).

In the subgroup of patients with low Cox-2 expression (using a cut-off IRS of 7), expression of cytoplasmic PPARγ was still related to a poor prognosis (p = 0.009, Fig. 3c) while no correlation of cytoplasmic PPARγ and OS existed for the patients with high Cox-2 expression (Fig. 3d).

### Cytoplasmic PPARγ expression as an independent prognostic parameter of OS in N-cadherin low and Cox-1 negative tumors

We then performed multivariate analyses for the whole cohort and for the subgroups of patients described above, using the Cox regression model with cytoplasmic PPARγ expression and various clinicopathological features (age at time of diagnosis, tumor size, ER, and HER2 status). As shown in Table 5, data demonstrated that in the whole cohort, only age, tumor size and ER were independent prognostic markers of OS. Very interestingly, cytoplasmic PPARγ appeared as an independent prognosis marker in the N-cadherin low (IRS < 3.5) and Cox-1 negative subgroups (p = 0.044 and p = 0.014 respectively), with hazard ratios of 1.996 and 2.047 indicating a much higher risk of death for the patients with tumors expressing high levels of cytoplasmic PPARγ.

**Table 5:** Multivariate analysis of significant clinicopathological variables and of cytoplasmic PPARγ regarding OS in the whole cohort and in various subgroups

	Age	pT	ER	HER2	Cytoplasmic PPARγ
Whole cohort					
p	0.00001***	0.00000007***	0.008**	0.154	0.129
HR	1.040	3.769	0.508	1.616	1.457
N-cadherin low					
p	0.002**	0.00037***	0.015*	0.528	0.044*
HR	1.041	3.370	0.420	1.341	1.996
N-cadherin high					
p	0.000733***	0.000032***	0.174	0.035*	0.902
HR	1.045	6.121	0.583	3.437	1.052
Cox-1 negative					
p	0.000023***	0.000008***	0.162	0.307	0.014*
HR	1.045	3.835	0.655	1.598	2.047
Cox-1 positive					
p	0.017*	0.017*	0.023*	0.253	0.454
HR	1.051	3.574	0.284	1.907	0.670
Cox-2 low					
p	0.000002***	0.00008***	0.015*	0.969	0.102
HR	1.058	3.272	0.440	0.983	1.665
Cox-2 high					
p	0.112	0.000343**	0.801	0.021*	0.545
HR	1.027	7.681	0.867	5.369	1.427

HR, hazard ratio; p, p-value

In the sub-groups, the same cut-off as in Figs. 2 and 3 have been used, namely 3.5 for N-Cadherin and 7 for Cox-2. Correlations are statistically significant for p < 0.05 (*) or p < 0.01 (**), p < 0.001 (***)

On the opposite, cytoplasmic PPARγ had no independent prognostic value in the N-cadherin high or Cox-1 positive expressing subgroups, in the subgroups with low or high Cox-2 expression (IRS cut-off of 7) or even in the Luminal A subgroup (data not shown). The same analysis performed with nuclear or total PPARγ, with Cox-1 or Cox-2, did not reveal any independent prognostic value as seen with cytoplasmic PPARγ.

### Cox-1 expression is associated with favorable OS only in tumors with high cytoplasmic PPARγ expression

We then checked in the whole cohort that neither Cox-1 nor Cox-2 expression was related to OS (Additional file 1: Figure S2A, B respectively). In order to strengthen the link between PPARγ, Cox1, and survival, we analyzed the prognostic value of Cox1 according to PPARγ levels. By selecting patients with tumors expressing high levels of cytoplasmic PPARγ (Fig. 3f), Cox-1 expression appeared statistically correlated to a better OS of patients (p = 0.032). For patients with tumors expressing low levels of cytoplasmic PPARγ (Fig. 3e), no correlation with OS appeared although we observed again an opposite trend, with Cox-1 expression numerically correlated with a poor OS. Altogether, these data strengthened our results demonstrating that the relative expression of cytoplasmic PPARγ and Cox-1 is linked to prognosis in primary BC, with a high cytoplasmic PPARγ/Cox-1 ratio being a marker for poor prognosis, and that Cox-1 expression correlated with longer OS in an unselected cohort.

## Discussion

The aim of this study was to characterize the intracellular expression and possible interplay of PPARγ and the Cox (Cox-1 and Cox-2) expression in a wide range of BC specimens, in relation with the clinicopathological parameters as well as patient survival. We already demonstrated that cytoplasmic PPARγ is overexpressed in BRCA1 mutated BC compared to sporadic cases, but without correlation to survival [27]. In previous surveys, either nuclear PPARγ or cytoplasmic PPARγ had a correlation with an improved clinical outcome of BC patients [8, 36, 37], but fewer specific subgroups of patients were analyzed.

Our data demonstrated that PPARγ expression was detected in a majority of BC tissues and that it is predominantly localized in cytoplasm (92.3% vs 18.7%). This is in accordance with previous studies [8, 27, 38]. However, positive PPARγ immunoreactivity was previously described as mainly nuclear in normal cells from benign samples; in malignant cells, a decreased expression was shown which was related to a favorable survival for patients [37, 39]. In addition, it was demonstrated that casein-kinase-II-dependent phosphorylation of PPARγ leads to subcellular translocation of PPARγ from cytoplasm to nucleus regulated by CRM1 and that urokinase-type plasminogen activator promoted atherogenesis in hepatocytes by downregulating PON1 gene expression via PPARγ nuclear export mechanism [9, 40]. Intracellular distribution of PPARγ was observed in BC tissues and cell lines [41], suggesting that poorly differentiated samples and highly invasive cell lines displayed mainly cytoplasmic PPARγ expression. Moreover, cytoplasmic localization of PPARγ was described as being mediated by Skp2 upon MEK1-dependent mechanism indicating cytoplasmic translocation of PPARγ promoted tumorigenesis in BC. In another study [17], α-ESA, considered as a PPARγ agonist like rosiglitazone, as well as GLA [38], suppressed cell growth in BC cell lines by activating PPARγ nuclear compartmentalization, which suggested that nuclear localization of PPARγ plays a role in anti-cancer functions in BC. Besides the predominant cytoplasmic localization of PPARγ, our data demonstrate a significant correlation between total and cytoplasmic PPARγ and an inverse relationship between cytoplasmic and nuclear PPARγ (Table 3), supporting the hypothesis of the translocation mechanism of PPARγ in the carcinogenic process.

Concerning the correlation between PPARγ expression and clinicopathological features or aggressiveness markers, our data demonstrated that nuclear PPARγ expression was inversely correlated with tumor grade, HER2 and N-cadherin expression, whereas total and cytoplasmic PPARγ were positively related with HER2, CD133, and N-cadherin (Fig. 1 and Table 4). These correlations strongly suggest that only cytoplasmic PPARγ was associated with the more aggressive tumors, namely ER negative, HER2 positive, CD133 (as a CSC marker [33, 34]) positive and NCAD (as an EMT marker [35]) positive sub-groups. Nonetheless, cytoplasmic PPARγ expression being much higher (15 fold) than nuclear one, total PPARγ expression exhibited similar association as cytoplasmic one with tumor aggressiveness. Several authors also found, as we did, a negative correlation between nuclear PPARγ and histological grade [36, 37, 39], and one paper indicated that nuclear PPARγ was negatively associated with HER2 [39]. Interestingly, PPARγ protein was expressed in both transfected MCF-7/Neo and MCF-7/HER2, but with higher levels of expression in the MCF-7/HER2 cells [42]. Moreover, HER2 up-regulated PPARγ expression, causing BC cells to become resistant to PPARγ ligand response [43]. Both CD133 and N-cadherin play a critical role in cancer migratory and invasive properties. Indomethacin could decrease CD133 expression, which means reducing CSCs via inhibiting Cox-2 and NOTCH/HES1 and activating PPARγ [44]. According to our previous work [29], N-cadherin-positive tumors without LNM had a significantly shorter survival time. Enhanced activity of PPARγ had an inhibition on TGF-β induction of N-cadherin promoter in lung carcinoma cell lines [45].

Overall, nuclear PPARγ possess a possible protective role against BC development, whereas cytoplasmic PPARγ was defined as a promoter during BC progression. Our data emphasize this hypothesis of opposite correlation of nuclear PPARγ with antioncogenic parameters and of cytoplasmic PPARγ with oncogenic or aggressive parameters. Survival analysis in the whole cohort demonstrated that only cytoplasmic PPARγ expression had a strong correlation with poor OS (Fig. 2), whereas both total and cytoplasmic PPARγ expression had a strong correlation with poor RFS (Additional file 1: Figure S1). As described earlier, PPARγ activation has been shown to exert antiproliferative and pro-apoptotic effects in BC cell lines [16–18, 46]. Moreover, cell death has been shown to be triggered in BC cell lines through the localization of PPARγ into the nucleus followed by the induction of Fas ligand [19]. The analysis of apoptosis markers will be necessary to give more insight in the molecular mechanisms underlying the differential effects of cytoplasmic and nuclear PPARγ.

Analysis of Cox in our cohort of primary BC substantiated that both Cox-1 and Cox-2 were dominantly localized in cytoplasm with a predominant negative or low expression for Cox-1 and a high expression for Cox-2 (Table 2). However, they were both significantly and positively correlated with total and cytoplasmic PPARγ, whereas only Cox-2 expression was negatively correlated with nuclear PPARγ (Table 3). Additionally, similarly to cytoplasmic PPARγ, Cox-1 was positively associated with HER2, CD133, and N-cadherin. Nonetheless, it was inversely related to LN involvement (Table 4), suggesting the hypothesis that Cox-1 expression may be related to the evolution of the tumor, especially expressed during the early non-metastatic stages of BC. Moreover, Cox-2 was positively related to Ki-67, CD133, and N-cadherin. In breast CSCs deprived from tumor cells of HER2/Neu mice, both Cox-1 and Cox-2 genes, belonging to a set of genes representing possible molecular targets correlated with BC survival, are overexpressed [47]. Compared to Cox-2, less attention was paid to Cox-1 in tumors and fewer data elucidated that Cox-1 selective inhibitors, such as SC-560 [48], catechin [49] and FR122047 [50], suppressed cell growth in BC. More interestingly, corticotropin-releasing factor, a hypothalamic neuropeptide, promoted cell invasiveness in MCF-7 BC cell line via induction of Cox-1 expression but not of Cox-2, as well as the production of prostaglandins [51].

Cox was officially known as an enzyme responsible for the synthesis of PGs from arachidonic acid. The role of Cox-2 and PPARγ in pro-apoptosis and tumor regression was explored in lung cancer cell lines, demonstrating that cannabidiol induced the upregulation of Cox-2 and PPARγ following a nuclear translocation of PPARγ by Cox-2 dependent PGs [52]. Modulation of 15d-PGJ_2_, a natural ligand of PPARγ, may influence the development of BC progress [53]. Cox-1 could lead to the activation of PPARγ [54]. Our finding of a strong correlation between Cox-1 and cytoplasmic PPARγ highlight their possible interaction in BC cells. Furthermore, Cox-1 and Cox-2 expression has been shown to be strongly associated in BC to the expression of the aromatase (CYP19A1) [55] which has been shown to be associated with a poor survival of ER positive BC patients [56]. As a consequence, the link of cytoplasmic PPARγ with poor survival might involve the dysregulation of CYP19A1 expression through Cox activity. Obviously, other mechanisms might participate and further work will be needed to decipher the precise underlying mechanisms.

In our study, although neither Cox-1 nor Cox-2 were related to OS in the whole cohort (Additional file 1: Figure S2A, B), high cytoplasmic PPARγ expression was significantly associated with poor OS in the Cox-1 negative subgroup and in the Cox-2 low expression subgroup (Fig. 3a, c). In addition, we also observed that the trend was inverted with an apparent, although not significant, better prognosis for the patients with high cytoplasmic PPARγ expression in the Cox-1 positive subgroup. Moreover, the data we generated demonstrate that cytoplasmic PPARγ expression is an independent prognostic marker in the Cox-1 negative subgroups, related to a twofold higher risk of death for those patients. Interestingly, positive Cox-1 expression (inversely related to the LN status) was defined as a favorable outcome marker for the patients with high cytoplasmic PPARγ expression (Fig. 3f), and tended to be a bad outcome marker for the patients with low cytoplasmic PPARγ expression. Our data suggest that the expression of Cox-1 and cytoplasmic PPARγ are interdependent, with the ability for Cox-1 to rescue the negative impact of cytoplasmic PPARγ on patient outcome. A hypothesis could be a potential role of Cox-1 in nucleocytoplasmic translocation of PPARγ, thereby suppressing tumor growth.

## Conclusions

In our primary BC cohort, PPARγ was predominantly expressed in cytoplasm of BC cells and may perform different roles in tumorigenesis according to its subcellular localization. Cytoplasmic PPARγ was strongly correlated with Cox-1 mainly, as well as with other bad prognosis markers (HER2, CD133, N-cadherin), contributing to explore their interactions during BC progression. High cytoplasmic PPARγ expression was correlated with short OS in the whole cohort and in several subgroups with good prognosis. A major conclusion is that this bad prognostic impact of cytoplasmic PPARγ depends on Cox-1 expression, as it is worse when Cox-1 is negative and lost when Cox-1 is expressed. Altogether, this leads to the strengthening that the intracellular PPARγ localization might be involved in tumorigenesis, and to the conclusion that cytoplasmic PPARγ may be defined as a potential therapeutic target and a prognostic marker in BC. Further analyses are now needed to decipher the molecular mechanisms underlying PPARγ interplay with Cox-1 and Cox-2 to modulate BC aggressiveness through the control of cell proliferation and/or apoptosis.

## Supplementary information

**Additional file 1: Figure S1.** Kaplan–Meier analysis in the whole cohort of patient overall survival according to Total PPARγ expression and patient relapse-free survival according to total, cytoplasmic and nuclear PPARγ expression. Overall survival (OS) curves are presented according to total PPARγ (A) status. Relapse-free survival (RFS) curves are presented according to total (B), cytoplasmic (C) and nuclear (D) PPARγ status. The IRS cut-off values with the number of cases for each group are indicated in each graph. Statistical significance is shown as p-value from log-rank test (*: p < 0.05; **: p < 0.01). **Figure S2.** Kaplan–Meier analysis in the whole cohort of patient overall survival according to Cox-1 or Cox-2 expression. Overall survival (OS) curves are presented according to Cox-1 (A) or Cox-2 expression. The IRS cut-off values with the number of cases for each group are indicated in each graph. Statistical significance is shown as p-value from log-rank test (*: p < 0.05; **: p < 0.01).

## Data Availability

All data generated or analysed during this study are included in this published article and its Additional file.

## Abbreviations

BC: Breast cancer
Cox: Cyclooxygenase
CSC: Cancer stem cell
DCIS: Ductal carcinoma in situ
EMT: Epithelial mesenchymal transition
ER: Estrogen receptor
FISH: Fluorescence in situ hybridization
HER2: Human epidermal growth factor receptor 2
HR: Hazard ratio
IHC: Immunohistochemistry
IRS: Immunoreactive score
LCoR: Ligand-dependent corepressor
LMU: Ludwig Maximilians University
LNM: Lymph node metastasis
NR: Nuclear receptor
NST: Non-special type
OS: Overall survival
PBS: Phosphate buffered saline
PG: Prostaglandin
pN: Primary lymph node
PPARs: Peroxisome proliferator-activated receptors
PPARγ: Peroxisome proliferator-activated receptor γ
PPREs: Proliferator-activated receptor response elements
PR: Progesterone receptor
pT: Primary tumor size
RAR: Retinoic acid receptor
RFS: Relapse-free survival
RIP140: Receptor interacting protein of 140 kDa
ROC-curve: Receiver operating characteristic curve
RXR: Retinoid X receptor
TNBC: Triple-negative breast cancer
TPA: Tetradecanoyl phorbol acetate
