# Supplementary material for: Cytoplasmic PPARγ is a marker of poor prognosis in patients with Cox-1 negative primary breast cancers
Source: J Transl Med. 2020 Feb 21;18:94. doi: 10.1186/s12967-020-02271-6 (PMC7035771; doi:10.1186/s12967-020-02271-6)
Supplement: Supplementary file 1 — Additional file 1: Figure S1. Kaplan–Meier analysis in the whole cohort of patient overall survival according to Total PPARγ expression and patient relapse-free survival according to total, cytoplasmic and nuclear PPARγ expression. Overall survival (OS) curves are presented according to total PPARγ (A) status. Relapse-free survival (RFS) curves are presented according to total (B), cytoplasmic (C) and nuclear (D) PPARγ status. The IRS cut-off values with the number of cases for each group are indicated in each graph. Statistical significance is shown as p-value from log-rank test (*: p < 0.05; **: p < 0.01). Figure S2. Kaplan–Meier analysis in the whole cohort of patient overall survival according to Cox-1 or Cox-2 expression. Overall survival (OS) curves are presented according to Cox-1 (A) or Cox-2 expression. The IRS cut-off values with the number of cases for each group are indicated in each graph. Statistical significance is shown as p-value from log-rank test (*: p < 0.05; **: p < 0.01). [file 12967_2020_2271_MOESM1_ESM.pptx]

## Slide 1
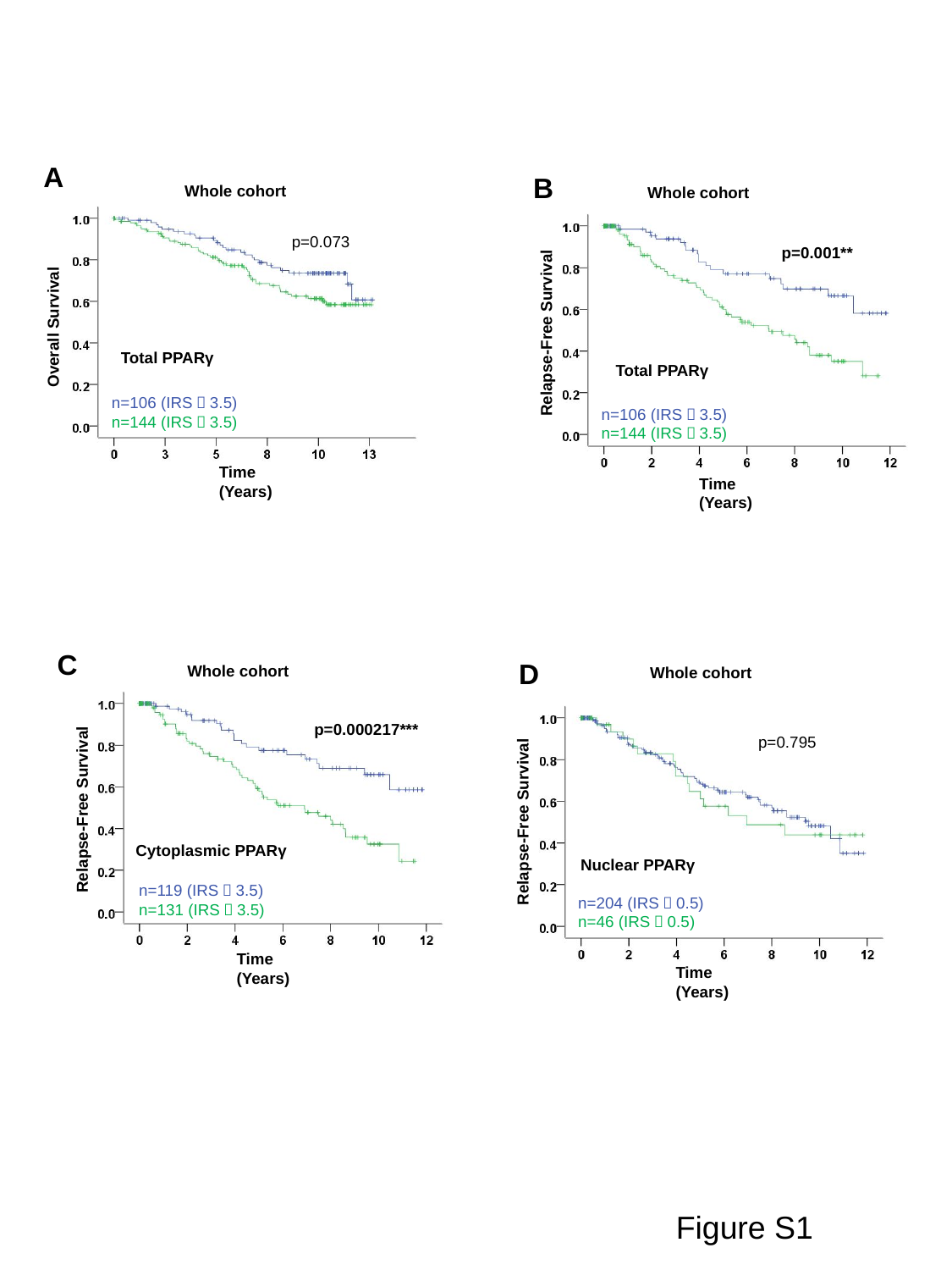

A
p=0.073
Overall Survival
Total PPARγ
n=106 (IRS＜3.5)
n=144 (IRS＞3.5)
Time (Years)
B
Whole cohort
Whole cohort
p=0.001**
Relapse-Free Survival
Total PPARγ
n=106 (IRS＜3.5)
n=144 (IRS＞3.5)
Time (Years)
C
D
Whole cohort
Whole cohort
p=0.000217***
p=0.795
Relapse-Free Survival
Relapse-Free Survival
Cytoplasmic PPARγ
Nuclear PPARγ
n=119 (IRS＜3.5)
n=131 (IRS＞3.5)
n=204 (IRS＜0.5)
n=46 (IRS＞0.5)
Time (Years)
Time (Years)
Figure S1

## Slide 2
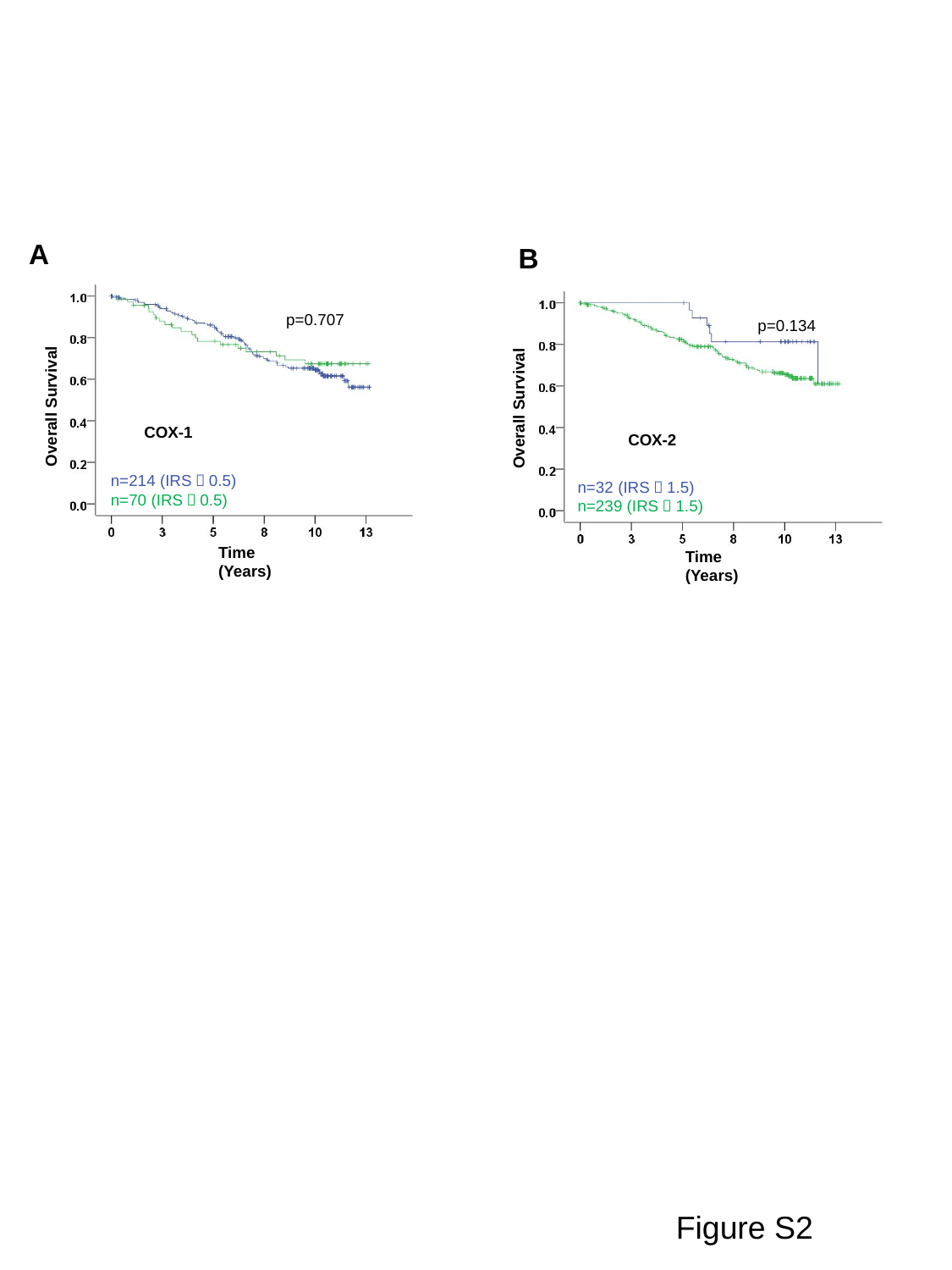

A
B
p=0.134
Overall Survival
n=32 (IRS＜1.5)
n=239 (IRS＞1.5)
Time (Years)
p=0.707
Overall Survival
COX-1
COX-2
n=214 (IRS＜0.5)
n=70 (IRS＞0.5)
Time (Years)
Figure S2
